# Research on the Purchase Behavior of Owner–Pet Matching Outfits Based on the Extended Theory of Planned Behavior

**DOI:** 10.3390/bs16061021

**Published:** 2026-06-18

**Authors:** Sisi Chen, Diqing Qian, Zengrui Xiao

**Affiliations:** 1 School of Fashion Design and Engineering, Zhejiang Sci-Tech University, Hangzhou 311106, China; 2024220402004@mails.zstu.edu.cn; 2 International Institute of Fashion Technology, Zhejiang Sci-Tech University, Hangzhou 311106, China

**Keywords:** owner–pet matching outfits, theory of planned behavior, purchase intention, purchase behavior, aesthetic risk

## Abstract

With the rapid expansion of the pet economy, owner–pet matching outfits have grown increasingly popular among pet owners. Grounded in the extended theory of planned behavior, this study investigates the key determinants of pet owners’ purchase intentions and actual purchase behaviors toward owner–pet matching outfits, and explores the moderating effect of aesthetic risk on the intention–behavior transition. Questionnaire survey data from 222 pet owners were collected for empirical analysis, and regression analysis was adopted to verify the proposed research hypotheses. The empirical results reveal that subjective norms exert a direct promotional effect on consumer purchase behavior and indirectly boost such behavior through the partial mediating role of purchase intention. By contrast, behavioral attitude is positively associated with purchase intention and further stimulates purchase behavior via a full mediating pathway of purchase intention. Perceived behavioral control displays a significant positive direct impact on purchase behavior yet yields no significant effect on purchase intention. Furthermore, purchase intention serves as a robust positive predictor of purchase behavior, whereas aesthetic risk significantly weakens the association between purchase intention and purchase behavior. Brands are suggested to foster consumers’ favorable behavioral attitudes by optimizing product design, enriching practical functions, and minimizing potential risks to pets in owner–pet matching outfits. Meanwhile, enterprises should actively shape supportive subjective norms to popularize the owner–pet matching outfit wearing lifestyle. Additionally, brands need to enhance consumption accessibility through diversified sales channels, reasonable pricing strategies and abundant product style options. This study pioneers the application of the extended theory of planned behavior to the emerging field of owner–pet matching outfits, empirically verifying the positive effects of behavioral attitude, subjective norms, and perceived behavioral control on consumers’ purchase intention and purchase behavior.

## 1. Introduction

With the rapid growth of pet populations worldwide, the prosperity of the pet economy has emerged as one of the most notable social trends, reflecting the profound transformation of social structures and the upgrading of emotional consumption on a global scale (Gao, 2025). Not only are basic pet consumption sectors thriving—including pet food (Ai et al., 2025), healthcare (Becker et al., 2022), and care services (Liang et al., 2025)—but the pet fashion market, encompassing pet apparel (Apaolaza et al., 2022), pet beauty services (McDonald et al., 2022), and pet accessories (Maidin et al., 2025), is also expanding at an accelerated pace. As a specialized extension of pet apparel, owner–pet matching outfits are also gaining popularity. Pets are increasingly endowed with the status of “fur babies”, serving as important sources of emotional comfort for young people. Owner–pet matching outfits not only embody pet owners’ care and affection for their pets but also further strengthens the emotional bond between owners and their animal companions (Apaolaza et al., 2022; Vänskä, 2016). Moreover, showcasing pets, dressing in matching outfits in many cases, has become a popular trend among pet owners and on social media (Zhang et al., 2023). However, existing research on pet fashion consumption has primarily focused on pet apparel in general, with few empirical studies specifically examining the purchase behavior of owner–pet matching outfits through a socio-psychological lens. So, what factors motivate or hinder owners’ purchase behavior of owner–pet matching outfits?

Currently, the owner–pet matching outfits industry is still in its nascent stage, with a relatively small market size and facing many doubts. The lack of well-known brands, imperfect manufacturing standards, incomplete size ranges, and limited distribution channels may impede consumers’ purchasing decisions (Gao, 2025). Prevailing unfavorable ethical beliefs on species differences and negative individual attitudes on owner–pet matching outfits also exert adverse impacts on consumers’ purchase intentions (D’Souza et al., 2023; Menor-Campos, 2024). It is also noteworthy that while some pet owners express high interest in these matching outfits, they often fail to translate this interest into actual purchase behavior (Apaolaza et al., 2022). Against this backdrop, the extended theory of planned behavior constitutes an appropriate analytical framework to unpack the effects and mechanisms of the determinants on pet owners’ actual purchase behavior. While the theory of planned behavior has been successfully applied to various fashion consumption contexts (Cuong, 2024; Y. Wang et al., 2024; Wei et al., 2025), its application to the emerging field of owner–pet matching outfits remains unexplored. Unlike conventional apparel consumption, owner–pet matching outfits are more likely to attract attention, involve dyadic decision-making (both owner and pet), heightened emotional attachment (Apaolaza et al., 2022), and unique aesthetic risk concerns (Rausch & Kopplin, 2021), which may alter the conventional pathway in the theory of planned behavior. This represents a critical gap in understanding the unique determinants of purchase behavior regarding owner–pet matching outfits and a potential to advance the theory of planned behavior.

To address this research gap, this study draws on the extended theory of planned behavior to explore the impacts of behavioral attitude, subjective norms, and perceived behavioral control on consumers’ purchase behavior of owner–pet matching outfits, with purchase intention serving as a mediating variable and aesthetic risk as a moderating variable. The empirical findings reveal that perceived behavioral control exerts a direct positive effect on pet owners’ purchase behavior; behavioral attitude promotes purchase behavior indirectly through the mediating role of purchase intention; and subjective norms influence purchase behavior both directly and partially through the partial mediation of purchase intention. Additionally, aesthetic risk significantly weakens the positive relationship between purchase intention and purchase behavior. This research combines the characteristics of pet fashion and emotional consumption, and revisits the classic model of theory of planned behavior to explore the unique consumption decision-making mechanism of owner–pet matching outfits, which can effectively supplement the existing pet economy and fashion consumption research.

## 2. Theories and Hypotheses

### 2.1. Theory of Planned Behavior

The theory of planned behavior is a robust socio-psychological model that effectively explains the formation of individual behavioral intentions and the mechanism by which these intentions are translated into specific actual behaviors (Batool et al., 2024; Laheri et al., 2024; C. Wang et al., 2025). Originating from the theory of reasoned action proposed by Fishbein and Ajzen (1975), the theory of planned behavior was further refined by Ajzen through the introduction of a new core variable: perceived behavioral control (Ajzen, 1985). The core logic of the theory of planned behavior holds that three key constructs—behavioral attitude, subjective norms, and perceived behavioral control—jointly influence an individual’s behavioral intention, which in turn leads to the performance of actual behavior (Ajzen, 1991).

Behavioral attitude, also referred to as attitude toward the behavior, denotes an individual’s positive or negative feelings and evaluative judgments toward a specific behavior, reflecting their inherent preference or aversion to engaging in that behavior (Laheri et al., 2024). It is closely associated with an individual’s expectations regarding the outcomes of performing the specific behavior (Ajzen, 1991): the more positive an individual’s expectations of the behavior’s outcomes, the more likely they are to hold a favorable attitude toward the behavior, and consequently, the more eager they will be to engage in it (Y. Wang et al., 2024). While behavioral attitude tends to be relatively enduring, it can undergo changes as a result of new experiences or the acquisition of new information relevant to the behavior.

Subjective norms refer to the social pressure an individual perceives when considering engaging in a specific behavior, which primarily stems from their understanding of the expectations of significant others (e.g., family, friends) or relevant social groups (Ajzen, 2020). When an individual perceives support or encouragement from these significant others and believes that their behavior aligns with the norms and expectations of the social group to which they belong, they are more inclined to adopt that behavior. Subjective norms also encompass the intensity of an individual’s willingness to conform to the expectations of these significant others and social groups (Liu et al., 2023); the more important these reference individuals or groups are to the individual, the greater the impact of subjective norms on their behavioral decisions.

Perceived behavioral control refers to an individual’s self-assessment of the difficulty or ease of executing a specific behavior (C. Wang et al., 2025). In practice, individuals often refrain from engaging in behaviors they favor or behaviors expected by social groups due to external obstacles (e.g., time constraints, financial limitations, or limited access) (Rahman et al., 2023). Conversely, when an individual perceives fewer obstacles to performing a certain behavior, their sense of control over that behavior becomes stronger, and they are correspondingly more likely to engage in it.

In addition to these three core factors, the theory of planned behavior is often extended by incorporating other relevant variables, especially contextual factors, to enhance its explanatory power and make the research conclusions more rigorous and applicable to specific research contexts (Qi & Ploeger, 2019; Si et al., 2022). This extended framework is particularly suitable for exploring complex consumption behaviors such as the purchase of owner–pet matching outfits, where multiple contextual and psychological factors interact to influence decision-making.

### 2.2. Behavioral Attitude

As an extension of the parent–child matching outfit concept, owner–pet matching outfits strengthen the emotional bond between humans and pets through resonance in colors, patterns, or styles. While this emerging fashion trend has gained traction, it is not universally accepted or adopted by all individuals. In the context of this study, behavioral attitude refers to consumers’ overall positive or negative evaluations of purchasing owner–pet matching outfits (Conradie et al., 2023; Ye & Kuang, 2026). It encompasses two distinct facets: instrumental attitude and affective attitude (Apaolaza et al., 2022; Fantechi et al., 2024). Instrumental attitude reflects consumers’ rational judgments of the practical attributes of the clothing, particularly whether it meets the physiological needs of both pets and themselves. For instance, some consumers perceive owner–pet matching outfits as aesthetically pleasing while also helping to keep pets warm and clean (Chung, 2023). Such individuals are likely to hold a positive instrumental attitude toward owner–pet matching outfits. Conversely, some individuals argue that pets do not require clothing, and improperly designed outfits may harm pets’ skin, restrict their movements, or even trigger anxious behaviors—leading to a negative instrumental attitude. Affective attitude, on the other hand, refers to whether consumers can derive positive emotional experiences and psychological satisfaction from the consumption behavior. Many consumers view owner–pet matching outfits as an expression of affection for their pets and a symbol of their unique lifestyle, thereby forming a positive affective attitude (Apaolaza et al., 2022; Vänskä, 2016). By dressing themselves and their pets in matching outfits, they can obtain positive emotional feedback from others in daily life and through social media sharing (Apaolaza et al., 2022). However, some may question the excessive anthropomorphism of animals, viewing owner–pet matching outfits as a trap of consumerism and developing a negative affective attitude (Albloushy & Hiller Connell, 2019; Genç & İnce, 2025). Consistent with research on other product categories, when consumers hold a more positive attitude toward owner–pet matching outfits, they tend to have a stronger purchase intention and are more likely to engage in actual purchase behavior (N. Kumar et al., 2022; C. Wang et al., 2025). Based on this reasoning, the following hypotheses are proposed:

**H1a.**  *Behavioral attitude positively influences consumers’ purchase intention regarding owner–pet matching outfits*.

**H1b.**  *Behavioral attitude positively influences consumers’ purchase behavior regarding owner–pet matching outfits*.

**H1c.**  *Purchase intention mediates the relationship between behavioral attitude and purchase behavior regarding owner–pet matching outfits*.

### 2.3. Subjective Norms

Consumers’ purchase intention and actual purchase behavior are not only shaped by their own attitudes but also significantly influenced by social pressure (Etuk et al., 2022; Zahid et al., 2023). Subjective norms refer to an individual’s perception of social pressure to perform or refrain from performing a particular behavior, which is based on their understanding of the expectations of significant others (e.g., family, friends, or peer groups) (Carfora et al., 2019; C. Wang et al., 2025). Subjective norms can be further divided into two distinct dimensions: descriptive norms and injunctive norms, each exerting a unique influence on individual behavioral decisions (Hu & Hu, 2025). Descriptive norms operate by providing behavioral cues that indicate which behaviors are prevalent or encouraged within a social group. In the context of owner–pet matching outfits, the opinions and behaviors of other pet owners around an individual usually have the most significant impact on their consumption decisions (Kretzler et al., 2022; Liu et al., 2024). Social groups composed of pet owners typically share similar backgrounds, habits, and values, and social media platforms are filled with posts and shares related to owner–pet matching outfits—including advertisements from pet clothing brands and content showcasing the joyful atmosphere of owners and pets wearing matching outfits. Furthermore, when some owners and their pets appear in matching outfits at offline pet gatherings, they provide clear behavioral cues that such outfits are popular within the group. This phenomenon can even drive the formation of a fashion trend, prompting others to imitate the behavior to better integrate into the pet owner social group. Essentially, this imitative behavior is a process of symbolic transmission and sharing. By engaging in this symbolic consumption (i.e., purchasing and wearing owner–pet matching outfits), individuals seek consistency with their peer group and consolidate their sense of group belonging (Feng et al., 2025; Gruber et al., 2024). In contrast, injunctive norms directly guide individuals’ behaviors through explicit social expectations, specifying which behaviors are socially acceptable or prohibited. Within pet owner social groups, there may be implicit or explicit consensus regarding owner–pet matching outfits; in some cases, key opinion leaders may even explicitly put forward opinions and suggestions about such products. When these injunctive norms encourage the use of owner–pet matching outfits, individuals are more inclined to purchase such products to gain recognition from the group. In summary, descriptive norms reduce resistance in consumers’ purchase decisions by demonstrating prevalent group behaviors, while injunctive norms enhance their purchase motivation through explicit social expectations. Conversely, the absence of positive descriptive norms for owner–pet matching outfits within a group, or the presence of injunctive norms that explicitly oppose such styles, will discourage consumers’ purchase intention and inhibit their actual purchase behavior. Based on this reasoning, the following hypotheses are proposed:

**H2a.**  *Subjective norms positively influence consumers’ purchase intention regarding owner–pet matching outfits*.

**H2b.**  *Subjective norms positively influence consumers’ purchase behavior regarding owner–pet matching outfits*.

**H2c.**  *Purchase intention mediates the relationship between subjective norms and purchase behavior regarding owner–pet matching outfits*.

### 2.4. Perceived Behavioral Control

In many cases, even if an individual holds a positive behavioral attitude and is supported by favorable subjective norms, they may still fail to engage in the intended behavior due to various constraints. Perceived behavioral control refers to consumers’ self-evaluation of the ease or difficulty of completing the purchase behavior of owner–pet matching outfits (Hwang & Kim, 2020; Yadav & Pathak, 2016). This construct originates from the comprehensive influence of an individual’s perception of their own abilities and the controllability of the external environment (Ajzen, 2020). In terms of the external environment, the owner–pet matching outfits industry is still in an immature stage, characterized by frequent product quality issues, lack of universal size standards, serious design plagiarism and homogenization, limited distribution channels, and weak brand awareness. These factors objectively create multiple obstacles that hinder consumers from purchasing owner–pet matching outfits, reducing their perceived control over the purchase process. In addition, the influence of perceived behavioral control may be further exacerbated during economic downturns. For example, during the 2008 global financial crisis and the COVID-19 pandemic, many pet owners faced financial constraints that led to reduced spending on non-essential pet products, including pet fashion (Dogbey et al., 2024; Muldoon & Williams, 2024). In terms of personal abilities, consumers may face uncertainties and concerns: they may worry about whether they can accurately identify the appropriate sizes, materials, and styles of owner–pet matching outfits that suit both themselves and their pets, and they may also have concerns about whether they have sufficient financial capacity to bear the related costs. Such worries can subjectively make consumers feel that it is difficult to find satisfactory owner–pet matching outfits, further weakening their perceived behavioral control (Cuong, 2024; Y. Wang et al., 2024). On the contrary, when consumers perceive a strong sense of control over the purchase process, i.e., when their internal capabilities (e.g., the ability to select suitable products, sufficient financial resources) and the external environment (e.g., mature market norms, convenient channels, reliable product quality) collaborate to provide support, they will develop a stronger purchase intention and are more likely to engage in the actual purchase behavior of owner–pet matching outfits. Based on this reasoning, the following hypotheses are proposed:

**H3a.**  *Perceived behavioral control positively influences consumers’ purchase intention regarding owner–pet matching outfits*.

**H3b.**  *Perceived behavioral control positively influences consumers’ purchase behavior regarding owner–pet matching outfits*.

**H3c.**  *Purchase intention mediates the relationship between perceived behavioral control and purchase behavior regarding owner–pet matching outfits*.

### 2.5. Aesthetic Risk

Purchase intention is widely recognized as an important predictor of actual purchase behavior in consumer behavior research. Specifically, purchase intention refers to consumers’ willingness and subjective inclination to purchase a specific product, while purchase behavior denotes the concrete action of translating this intention into practical consumption behavior (B. Kumar et al., 2017; Lao, 2014). In general, the higher consumers’ purchase willingness toward a product, the greater the likelihood that this intention will be converted into actual purchase behavior. However, a certain gap often exists between purchase intention and actual purchase behavior in practice (Kaur & Bhardwaj, 2021; Frommeyer et al., 2022). This intention–behavior gap may stem from various constraints, including price fluctuations, perceived value discrepancies, emotional factors, and various types of perceived risks (Rausch & Kopplin, 2021; Diddi et al., 2019). In the field of fashion consumption, in particular, the formation of this gap is closely associated with aesthetic risk—a key contextual factor that has been increasingly emphasized in recent consumer behavior studies.

Aesthetic risk refers to consumers’ potential dissatisfaction or uncertainty regarding the aesthetic attributes of clothing (e.g., style, color, fit) before making a purchase (Rausch & Kopplin, 2021). Consumers typically care deeply about whether the clothes they purchase are aesthetically pleasing and whether they align with their self-image. For pet owners, this concern is further amplified: pets are often regarded as an extension of their self-identity, with owners projecting their own personalities onto their pets and paying close attention to their pets’ characteristics, behaviors, and appearance. When both owners and their pets appear in social situations wearing exquisite and fashionable matching outfits, they usually attract attention and spark social discussions, which in turn enhances the owner’s social image (Genç & İnce, 2025). As such, owner–pet matching outfits serve as a highly individualistic and expressive medium for pet owners to maintain their social image and convey their aesthetic preferences (Xia et al., 2023). However, when consumers are uncertain whether the owner–pet matching outfits can meet their aesthetic expectations, they may experience anxiety during the purchase decision-making process. The fear of receiving negative evaluations from others—such as being perceived as having poor taste—further weakens their determination to make a purchase (Kim et al., 2021). In this sense, aesthetic risk creates a psychological barrier between purchase intention and actual purchase behavior. Even if consumers initially hold a strong intention to purchase owner–pet matching outfits, excessive concerns about aesthetic risk may override their purchasing desires, inhibiting the conversion of intention into behavior. Therefore, the positive relationship between purchase intention and purchase behavior of owner–pet matching outfits will be weakened under conditions of higher aesthetic risk. Based on this reasoning, the following hypotheses are proposed:

**H4.**  *Purchase intention positively influences consumers’ purchase behavior regarding owner–pet matching outfits*.

**H5.**  *Aesthetic risk weakens the relationship between purchase intention and purchase behavior regarding owner–pet matching outfits, such that the relationship is weaker when the aesthetic risk is higher*.

**Figure 1:**
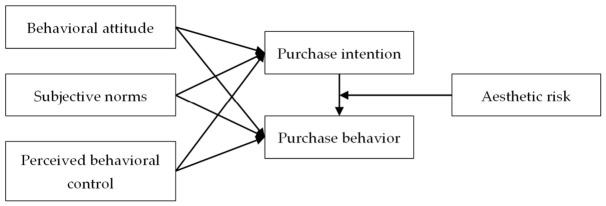
Theoretical framework and hypotheses. Source: Authors’ own elaboration based on Ajzen (1991).

The theoretical framework and hypotheses are shown in Figure 1.

## 3. Methodology

### 3.1. Samples and Procedures

To test the proposed hypotheses and achieve the research objectives, this study adopted a questionnaire survey method to collect empirical data. The survey was conducted from January to March 2026, spanning approximately three months. A combination of offline and online survey methods was employed to ensure diversity and representativeness. Specifically, researchers distributed paper questionnaires directly to pet owners at pet expos and pet-friendly parks in Hangzhou, while online questionnaires were disseminated through social media platforms such as RedNote (a Chinese APP similar to Instagram). A total of 251 questionnaires were distributed, and after rigorous screening to eliminate invalid responses (e.g., incomplete answers, inconsistent responses, and random filling), 222 valid samples were retained, resulting in an effective response rate of 88.4%—a rate that meets the requirements of empirical research in consumer behavior.

The demographic and pet-related characteristics of the valid samples are presented as follows. In terms of gender, 197 respondents were female, accounting for 88.7%, while 25 were male, accounting for 11.3%. The sample exhibits a notable gender imbalance; while this limits generalizability to male consumers, it is consistent with the target market of this product category. It reflects the demographic reality that pet fashion consumption, particularly owner–pet matching outfits, is predominantly driven by young women in China. Regarding age distribution, 99 respondents (44.6%) were aged between 18 and 25 years, 113 respondents (50.9%) were aged between 26 and 35 years, and only a small number of respondents were over 35 years old. In terms of education level, 144 respondents (64.9%) had a bachelor’s degree or above. With respect to monthly income, 87 respondents (39.2%) had a monthly income ranging from ¥4001 to ¥7000, and 45 respondents (20.3%) had a monthly income ranging from ¥7000 to ¥10,000. In terms of relationship status, 106 respondents (47.7%) were single, and 116 respondents (52.3%) were in a romantic relationship. Regarding fertility status, 200 respondents (90.1%) had no children. As for the types of pets kept, dogs accounted for the largest proportion, with 156 respondents (70.3%) keeping dogs. This is explainable given that dressing dogs is more feasible than dressing cats, rabbits, or birds due to dogs’ larger body size, greater tolerance for clothing, and higher frequency of outdoor activities.

### 3.2. Measures

As this study has applied the theory of planned behavior to the new research object of owner–pet matching outfits, the scales were adapted from previous research to fit the unique research context. The items of purchase behavior were measured by respondents’ actual purchase frequency or percentage, while the other items were measured with degree of agreement using a five-point Likert scale, where 1 represents “strongly disagree” and 5 represents “strongly agree”.

Behavioral Attitude. Behavioral attitude was measured with four items adapted from Conradie et al. (2023) and Ye and Kuang (2026). The items include “Wearing owner-pet matching outfits makes me look good”, “Wearing owner-pet matching outfits enhances my self-image”, “Wearing owner-pet matching outfits facilitates my social interactions”, and “Wearing owner-pet matching outfits showcases my close bond with my pet”. The Cronbach’s Alpha was 0.83.

Subjective norms. Subjective norms were measured with three items adapted from Carfora et al. (2019) and C. Wang et al. (2025). The items include “My pet-owning friends wear owner-pet matching outfits”, “Many internet celebrities share promotional contents about owner-pet matching outfits on social media”, and “People around me generally have a positive evaluation of wearing owner-pet matching outfits”. The Cronbach’s Alpha was 0.72.

Perceived behavioral control. Perceived behavioral control was measured with four items adapted from Hwang and Kim (2020) and Yadav and Pathak (2016). The items include “I can easily get access to owner-pet matching outfits”, “There are multiple channels for purchasing owner-pet matching outfits”, “Owner-pet matching outfits offers a wide variety of styles to choose from”, and “I can easily find owner-pet matching outfits that suit me and my pet”. The Cronbach’s Alpha was 0.90.

Aesthetic risk. Aesthetic risk was measured with four items adapted from Rausch and Kopplin (2021). The items include “I am concerned that the owner-pet matching outfits may not meet my aesthetic needs”, “I am concerned that the design and style of owner-pet matching outfits are not fashionable”, “When choosing owner-pet matching outfits, I am worried about buying designs and styles that don’t look good”, and “I am worried that many owner-pet matching outfits do not match my fashion taste”. The Cronbach’s Alpha was 0.89.

Purchase intention. Purchase intention was measured with four items adapted from B. Kumar et al. (2017). The items include “I am willing to purchase owner-pet matching outfits”, “I am likely to purchase owner-pet matching outfits in the future”, “I plan to purchase owner-pet matching outfits in the future”, and “I would recommend owner-pet matching outfits to my pet-owning friends”. The Cronbach’s Alpha was 0.90.

Purchase behavior. Purchase behavior was measured with three items adapted from Lao (2014). The items include “What is your frequency of purchasing owner-pet matching outfits in the past year”, “What percentage of your total pet clothing purchased (by quantity) in the past year consisted of owner-pet matching outfits?”, and “What percentage of your total expenditure on pet clothing in the past year was spent on owner-pet matching outfits?” The Cronbach’s Alpha was 0.87.

Control variables. Gender, age, education, monthly income, relationship status, fertility status, and types of pets were chosen as control variables.

## 4. Results

### 4.1. Validity Issues

Confirmatory factor analysis was conducted with the items of all variables to examine the validities of the measurement scales. The chi-square (χ^2^) of the model is 370.342, the degrees of freedom (DF) is 194, and the χ^2^/DF is 1.909, which is lower than the threshold value of 3. The root mean square error of approximation (RMSEA) is 0.064, which is lower than the threshold value of 0.08 and indicates that the covariance residuals are effectively controlled. The relative fit indices, such as the Tucker–Lewis index (TLI) is 0.927, the comparative fit index (CFI) is 0.939, and the incremental fit index (IFI) is 0.940; all exceed the threshold value of 0.9. All the indicators meet the statistical standards, indicating high consistency between the theoretical framework and the observed data.

Convergent validity. Convergent validity examines the strength of association among the items under the same latent variable. The results are shown in Table 1. All the standardized factor weights (λ) are higher than or very close to 0.7, the Composite Reliability (CR) is higher than or very close to 0.8, and the Average Variance Extracted (AVE) is higher than or very close to 0.5, indicating good convergent validity for the variables.

Discriminant Validity. Discriminant validity confirms the independence between different latent variables. The results are shown in Table 2. The square roots of AVE for all variables were greater than the correlation coefficients between that variable and other variables, which indicates a good discriminant validity.

### 4.2. Correlation Analysis

As shown in Table 2, purchase intention was positively correlated with behavioral attitude (r = 0.559, *p* < 0.01) and subjective norms (r = 0.486, *p* < 0.01), but not perceived behavioral control (r = 0.084, *p* > 0.05). Purchase behavior was positively correlated with behavioral attitude (r = 0.299, *p* < 0.01), subjective norms (r = 0.459, *p* < 0.01), and perceived behavioral control (r = 0.334, *p* < 0.01). In addition, purchase behavior was positively correlated with purchase intention (r = 0.399, *p* < 0.01). Apart from the relationship between purchase intention and perceived behavioral control, the other relationships are consistent with the hypotheses.

### 4.3. Hypotheses Testing

Hypotheses were tested with regression analysis. As shown in Table 3, four regression models were established to identify key factors influencing the purchase behavior of owner–pet matching outfits, using behavioral attitude, subjective norms, and perceived behavioral control as the independent variables, including the mediating variable purchase intention and the moderating variable aesthetic risk.

As shown in Model 1, purchase intention was significantly and positively associated with behavioral attitude (β = 0.411, *p* < 0.001) and subjective norms (β = 0.296, *p* < 0.001), but not perceived behavioral control (β = −0.064, *p* > 0.05). Thus, hypotheses H1a and H2a were supported, while hypothesis H3a was not supported.

As shown in Model 2, purchase behavior was significantly and positively associated with subjective norms (β = 0.323, *p* < 0.001) and perceived behavioral control (β = 0.185, *p* < 0.01), but not behavioral attitude (β = 108, *p* > 0.05). Thus, hypotheses H2b and H3b were supported, while hypothesis H1b was not supported.

As shown in Model 3, purchase behavior was significantly and positively associated with purchase intention (β = 0.268, *p* < 0.001), thus hypothesis H4 was supported. The mediating effects of purchase intention were further examined with a Sobel test, and the results are reported in Table 4.

As shown in Table 4, there is a significant mediating effect of purchase intention in the relationship between behavioral attitude and purchase behavior (a × b = 0.110, *p* < 0.01); thus, hypothesis H1c was supported. There is also a significant mediating effect of purchase intention in the relationship between subjective norms and purchase behavior (a × b = 0.079, *p* < 0.05); thus, hypothesis H2c was also supported. However, the mediating effect of purchase intention in the relationship between perceived behavioral control and purchase behavior was not significant (a × b = −0.017, *p* > 0.05); thus, hypothesis H3c was not supported.

**Figure 2:**
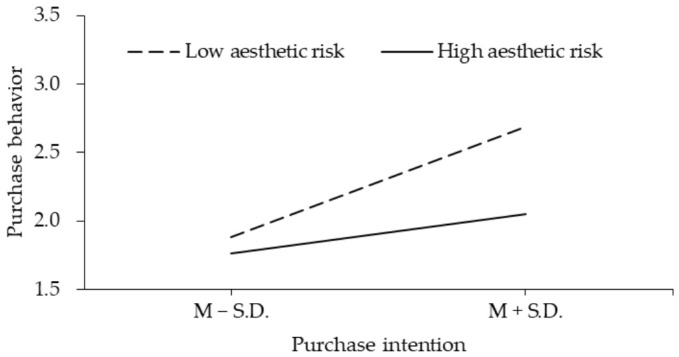
The moderating effect of aesthetic risk on the relationship between purchase intention and purchase behavior of owner–pet matching outfits. Source: Authors’ own analysis based on regression results.

As shown in Model 4 in Table 3, purchase behavior was significantly and negatively associated with the interaction term of purchase intention and aesthetic risk, which implied that aesthetic risk weakens the relationship between purchase intention and purchase behavior. To illuminate the moderating effect more clearly, the samples were separated into a low-aesthetic-risk group (M − S.D.) and a high-aesthetic-risk group (M + S.D.) for regression analysis. As shown in Figure 2, the positive relationship between purchase intention and purchase behavior is weaker at higher levels of aesthetic risk. Thus, hypothesis H5 was supported.

## 5. Discussion

### 5.1. Conclusions and Theoretical Contributions

This study aimed to examine the key factors determining pet owners’ purchase behavior of owner–pet matching outfits, based on the extended theory of planned behavior. Consistent with prior applications of theory of planned behavior (N. Kumar et al., 2022; C. Wang et al., 2025), this study confirms that subjective norms not only directly promote purchase behavior but also indirectly drive it through the mediating role of purchase intention. Existing studies have widely verified that behavioral attitude can exert both direct and indirect effects on actual purchase behavior (Apaolaza et al., 2022; Rausch & Kopplin, 2021). However, unlike these studies where behavioral attitude also directly predicts purchase behavior, our finding that the direct effect is non-significant suggests that in the owner–pet matching outfits context, behavioral attitude alone is insufficient to trigger action, and purchase intention serves as a necessary mediator. This difference may stem from the fact that owner–pet matching outfits are a symbolic and emotion-oriented fashion product, rather than a rigid daily necessity. Furthermore, unlike the conventional claims of theory of planned behavior where perceived behavioral control influences both intention and behavior (Hwang & Kim, 2020; Yadav & Pathak, 2016), our results show that perceived behavioral control does not significantly predict purchase intention but directly predicts purchase behavior. In mature consumer markets, perceived difficulty of purchase will leave a negative effect on consumers and change their willingness to buy. In contrast, the industry of owner–pet matching outfits remains in its infancy and features notable market imperfections, yet consumers show higher tolerance toward these issues. These objective external constraints do not necessarily undermine pet owners’ purchase intention, but they serve as the main barriers to actual consumption. This explains why perceived behavioral control exerts a direct effect on purchase behavior rather than purchase intention. This finding enriches the theory of planned behavior literature by revealing a context-specific pathway where perceived behavioral control bypasses intention. Additionally, consistent with the findings in sustainable clothing consumption (Rausch & Kopplin, 2021), when consumers perceive high aesthetic risk in owner–pet matching outfits, the positive relationship between purchase intention and actual purchase behavior is significantly weakened. Compared with general apparel, owner–pet matching outfits need to take both the owner’s and pet’s aesthetic and wearing experience into account, so consumers’ sensitivity to aesthetic risk is higher. This finding further verifies the applicability of aesthetic risk theory in segmented pet fashion consumption scenarios.

Based on these empirical conclusions, this study makes three distinct contributions to the existing literature and theory. First, in terms of research perspective and empirical evidence, it pioneers the application of the extended theory of planned behavior to the emerging field of owner–pet matching outfits, empirically verifying the positive effects of behavioral attitude, subjective norms, and perceived behavioral control on consumers’ purchase intention and purchase behavior. These findings are original in the research field of owner–pet matching outfits, filling the existing research gap and providing a theoretical foundation for further exploration of the antecedents of consumers’ purchase behavior in this specific context. Second, in terms of theoretical expansion of theory of planned behavior, this study reveals the mediating mechanism underlying the effects of behavioral attitude and subjective norms on purchase behavior: both constructs influence purchase behavior indirectly through the mediating role of purchase intention. This finding further clarifies the internal mechanism by which these constructs affect consumer behavior. Meanwhile, the study also finds that the mediating effect of purchase intention in the relationship between perceived behavioral control and purchase behavior is not significant, a result that differs from the conventional applications of the theory of planned behavior in other consumption fields, which enriches and deepens our understanding of the theory of planned behavior across different consumption contexts. Third, in terms of aesthetic risk perception research, this study identifies aesthetic risk as a key moderating variable that weakens the positive effect of purchase intention on purchase behavior. This finding explains the existing disconnect between purchase intention and actual purchase behavior in the context of owner–pet matching outfits, extends the extended theory of planned behavior by incorporating a context-specific moderating factor, and provides new insights into the boundary conditions of the relationship between purchase intention and behavior in fashion and pet consumption.

### 5.2. Practical Implications

Beyond the theoretical contributions, the empirical conclusions of this study also provide actionable practical inspirations for brands and practitioners in the owner–pet matching outfits industry. First, the findings indicate that consumers’ behavioral attitude is a critical antecedent of their purchase intention, yet many pet owners refrain from purchasing owner–pet matching outfits due to a lack of positive recognition of such products. To address this, brands should focus on optimizing product design, enriching practical functions, and minimizing potential harms to pets (e.g., using skin-friendly materials, designing pet-friendly cuts that do not restrict movement). These measures can help shape a positive behavioral attitude toward owner–pet matching outfits among consumers, thereby enhancing their purchase intention. Second, subjective norms exert both direct and indirect impacts on purchase behavior, and many consumers are deterred from purchasing owner–pet matching outfits due to concerns about social pressure or public prejudice. Brands should actively guide social norms by promoting the owner–pet matching lifestyle through diverse channels—including targeted advertisements, social media content creation, industry expos, and offline pet community activities. These efforts can help reduce public misunderstandings and prejudices toward owner–pet matching outfits, fostering supportive subjective norms that encourage the adoption of such products. Third, perceived behavioral control directly influences purchase behavior, and objective constraints such as limited distribution channels, inflated prices, and insufficient product styles have become key barriers to consumption. Brands should take targeted measures to address these obstacles: enrich their product lines to cover diverse styles and size ranges, formulate more reasonable pricing strategies that align with consumer purchasing power, and expand distribution channels (e.g., online e-commerce platforms, offline pet stores, and brand flagship stores). Fourth, given that aesthetic risk weakens the conversion of purchase intention to purchase behavior, and owner–pet matching outfits belong to the fashion consumption category, consumers pay close attention to the aesthetic appeal of both the pet’s and their own attire. Brands should prioritize the aesthetic design of owner–pet matching outfits, ensuring coordination in colors, patterns, and styles between the owner’s and pet’s clothing. Additionally, in promotional materials, brands should fully highlight the matching effect of the outfits on both owners and pets, helping consumers visualize the aesthetic value of the products and alleviate their concerns about aesthetic risk.

### 5.3. Limitations and Future Research

Despite the theoretical and practical contributions outlined above, this study has several limitations that should be acknowledged, providing directions for future research to address and expand upon. First, the sample size of the questionnaire survey in this study is relatively small, and the sample structure is relatively homogeneous: young female respondents constitute the main group, and the types of pets covered are primarily dogs. Although this sample distribution can be partially explained by the unique characteristics of the research object (i.e., owner–pet matching outfits, which are more popular among young female pet owners who keep dogs), it still limits the generalizability of the research findings. Dog owners typically walk their pets at least twice daily, increasing their exposure to social environments where owner–pet matching outfits are visible, which may amplify the effect of subjective norms. However, outfits may also be used for cats, rabbits, birds, or even fish, which could be even more sensitive to wearing clothing or accessories. In addition, purchase behavior of such outfits may also differ in different seasons, especially during extremely hot weather conditions. Future studies should expand the sample size and diversify the sample structure—including more male respondents, different age groups, owners of various pet types (e.g., cats, rabbits), and be conducted in different seasons—to examine whether these differences moderate pathways in the theory of planned behavior and enhance the robustness and external validity of the conclusions. Second, this study was conducted exclusively in China, a context where cultural values, social norms, and consumer perceptions regarding pets, dressing, and consumption may differ significantly from those in Western countries. For instance, consumers in China—a country with a high-collectivism cultural orientation—may attach strong importance to subjective norms when making purchase decisions, whereas this effect may be less pronounced among consumers in low-collectivism countries. Given the global expansion of the pet economy, future cross-cultural research is necessary to explore whether the relationships identified in this study hold across different cultural contexts, which will provide more comprehensive and universal insights for the global pet fashion industry. Future studies should also incorporate cross-cultural comparisons, particularly with other Asian countries (e.g., Japan, South Korea) and Western countries (e.g., America, France) to extend the generalizability of the conclusions. Third, this study focuses on exploring the antecedents of consumers’ purchase behavior of owner–pet matching outfits based on the extended theory of planned behavior, which provides a solid theoretical framework but does not cover all potential influencing factors. Other factors—such as perceived value (e.g., functional value, emotional value, social value), information processing modes, and social comparison tendencies—may also exert significant impacts on pet owners’ purchase decisions. Future research can draw on other relevant theories, such as the perceived value theory, information processing theory, and social comparison theory, to conduct more in-depth and diverse explorations, further enriching the understanding of consumer behavior in the context of owner–pet matching outfits.

## Figures and Tables

**Table 1:** Results of validity analysis.

Variables	Items	λ	CR	AVE
Behavioral Attitude	BA1	0.849	0.839	0.575
	BA2	0.897		
	BA3	0.717		
	BA4	0.509		
Subjective Norms	SN1	0.781	0.732	0.482
	SN2	0.731		
	SN3	0.548		
Perceived Behavioral Control	BC1	0.849	0.896	0.683
	BC2	0.842		
	BC3	0.827		
	BC4	0.787		
Aesthetic Risk	AR1	0.773	0.892	0.674
	AR2	0.899		
	AR3	0.836		
	AR4	0.770		
Purchase Intention	PI1	0.847	0.898	0.689
	PI2	0.821		
	PI3	0.855		
	PI4	0.795		
Purchase Behavior	PB1	0.722	0.877	0.707
	PB2	0.935		
	PB3	0.851		

Source: Authors’ own analysis.

**Table 2:** Correlations and square roots of AVE.

Variables	1	2	3	4	5	6
1. Behavioral Attitude	(0.758)					
2. Subjective Norms	0.529 **	(0.694)				
3. Perceived Behavioral Control	0.117	0.0367 **	(0.826)			
4. Aesthetic Risk	−0.137 *	−0.0161 *	−0.294 **	(0.821)		
5. Purchase Intention	0.559 **	0.486 **	0.084	−0.061	(0.830)	
6. Purchase Behavior	0.299 **	0.459 **	0.334 **	−0.317 **	0.399 **	(0.841)

Notes: * *p* < 0.05 (two-tailed); ** *p* < 0.01 (two-tailed). The square roots of AVE were displayed on the diagonal in brackets. Source: Authors’ own analysis.

**Table 3:** Results of regression analysis.

Variables	Purchase Intention	Purchase Behavior		
	Model 1	Model 2	Model 3	Model 4
Control variables				
Gender	−0.015	0.048	0.052	0.060
Age	−0.046	0.007	0.020	0.005
Education	−0.026	0.048	0.055	0.083
Monthly income	−0.029	−0.093	−0.085	−0.054
Relationship status	−0.018	−0.017	−0.012	−0.030
Fertility status	−0.054	−0.011	0.004	−0.009
Pets	0.006	−0.018	−0.020	−0.029
Independent variable				
Behavioral attitude	0.411 ***	0.108	−0.002	−0.018
Subjective norms	0.296 ***	0.323 ***	0.243 **	0.253 **
Perceived behavioral control	−0.064	0.185 **	0.203 **	0.137 *
Mediating variable				
Purchase intention			0.268 ***	0.289 ***
Moderating variable				
Aesthetic risk				−0.201 **
Interaction term				
Purchase intention × Aesthetic risk				−0.136 *
R^2^	0.378	0.261	0.306	0.367
F_(df1,df2)_	12.836_(10,211)_ ***	7.451_(10,211)_ ***	8.399_(11,210)_ ***	9.286_(13,208)_ ***

Notes: Standardized regression coefficients are reported; * *p* < 0.05; ** *p* < 0.01; *** *p* < 0.001. Source: Authors’ own analysis.

**Table 4:** Results of mediating effect analysis.

Path	a	Sa	b	Sb	a × b	z Value	*p* Value
BA→PI→PB	0.411	0.065	0.268	0.094	0.110	2.599	*p* < 0.01
SN→PI→PB	0.296	0.057	0.268	0.094	0.079	2.499	*p* < 0.05
BC→PI→PB	−0.064	0.041	0.268	0.094	−0.017	−1.369	*p* > 0.05

Source: Authors’ own analysis.

## Data Availability

Data is available on request from the authors.
